# Epigenetic Regulation of Virulence Gene Expression in Parasitic Protozoa

**DOI:** 10.1016/j.chom.2016.04.020

**Published:** 2016-05-11

**Authors:** Manoj T. Duraisingh, David Horn

**Affiliations:** 1 Department of Immunology and Infectious Diseases, Harvard T. H. Chan School of Public Health, 651 Huntington Avenue, Boston, MA 02115, USA; 2 Division of Biological Chemistry & Drug Discovery, School of Life Sciences, University of Dundee, Dow Street, Dundee DD1 5EH, UK

## Abstract

Protozoan parasites colonize numerous metazoan hosts and insect vectors through their life cycles, with the need to respond quickly and reversibly while encountering diverse and often hostile ecological niches. To succeed, parasites must also persist within individuals until transmission between hosts is achieved. Several parasitic protozoa cause a huge burden of disease in humans and livestock, and here we focus on the parasites that cause malaria and African trypanosomiasis. Efforts to understand how these pathogens adapt to survive in varied host environments, cause disease, and transmit between hosts have revealed a wealth of epigenetic phenomena. Epigenetic switching mechanisms appear to be ideally suited for the regulation of clonal antigenic variation underlying successful parasitism. We review the molecular players and complex mechanistic layers that mediate the epigenetic regulation of virulence gene expression. Understanding epigenetic processes will aid the development of antiparasitic therapeutics.

## Teaser

Protozoan parasites colonize numerous hosts with different environments and must tightly control gene expression to ensure survival and transmission. Duraisingh and Horn review the molecular players and complex mechanistic layers mediating the epigenetic regulation of clonal antigenic variation and virulence gene expression in the parasites causing malaria and sleeping sickness.

## Main Text

### Introduction

Protozoan parasites demonstrate varied and complex life cycles as they are transmitted within susceptible vertebrate populations. The diverse ecologies that parasites encounter have resulted in the evolution of significant species-specific elaborations for survival and propagation. A large number of these elaborations are epigenetic phenomena mediated by molecular determinants encoded by multigene families that can be highly diversified and variantly expressed.

Now that we are armed with a vast number of parasite genomes, it is clear that virulence genes often belong to multi-gene families that are greatly expanded, often numbering hundreds to thousands of genes, and representing an incredible commitment of resources in each genome. The vast majority of these virulence-related genes are found in specialized regions of protozoan chromosomes, typically the subtelomeric regions, facilitating both their variant expression and diversification through recombination. These “variantomes,” all of the genes that vary clonally in expression within a parasite, can encode for cell surface antigens, of which a few can act as adhesins to facilitate host cell attachment. Variant expression facilitates immune evasion and the colonization of different niches. Indeed, parasitic antigenic variation within vertebrate hosts has long been recognized as a key survival strategy, providing a mechanism for persistence in the face of host immune attack, by parasites as diverse as those that cause malaria, African trypanosomiasis and amebic dysentery.

In this review, we explore our emerging understanding of the epigenetic molecular mechanisms used by parasites to survive, transmit, thrive, and ultimately result in pathogenesis. We focus on two parasites, *Plasmodium falciparum* and *Trypanosoma brucei*, because they present evolutionarily distant organisms that have evolved disparate strategies for persistence in vertebrate hosts and have been the focus of considerable study. While the antigenic molecules themselves are completely unique to each parasite, the epigenetic regulators demonstrate many conserved features between protozoan parasites and model organisms. We will illustrate the common challenges of parasitism and highlight the varied solutions that these parasites have developed. However, it is now becoming clear that epigenetic control can also regulate other aspects of the parasitic life cycle, including the control of the switch from proliferative to developmental programs and the adaptation required for host and cellular tropism in different host niches.

### Parasite Persistence through Antigenic Variation

Malaria is caused by infection of vertebrate hosts by *Plasmodium* parasites that belong to the phylum apicomplexa and are transmitted between hosts by mosquitoes. The parasite goes through multiple morphological transformations in their vertebrate hosts as the sporozoite form first colonizes liver cells to form hepatic schizonts and merozoites that then infect red blood cells (RBCs). In RBCs, most parasites replicate in asexual cycles of proliferation. While in their human hosts, malaria parasites cause significant pathology following considerable proliferation and increase in parasite biomass. A few parasites become sexual forms that are subsequently taken up by the Anopheline mosquito vector for further transmission to humans.

**Figure 1:**
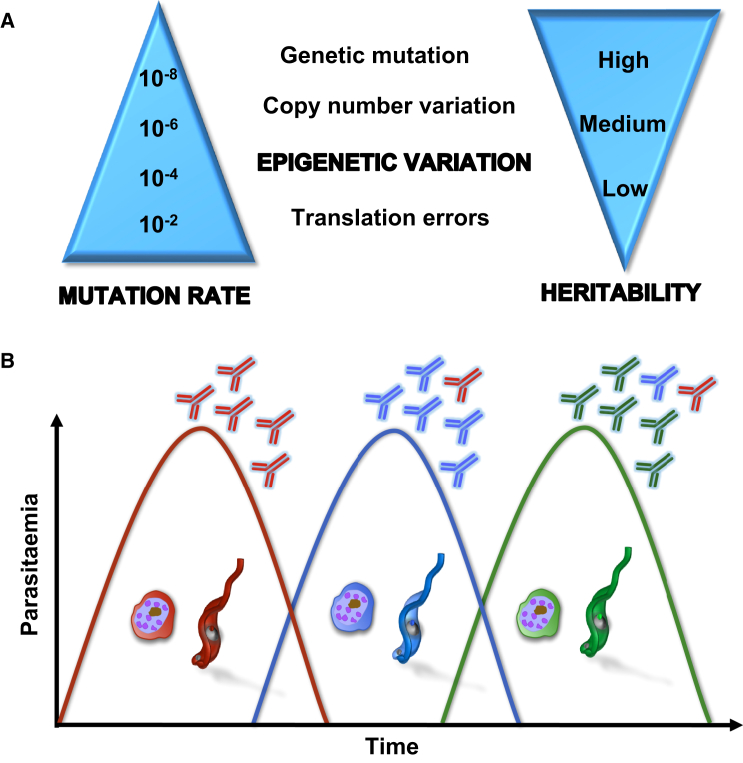
The Frequency of Epigenetic Variation Is Suitable for Driving Antigenic Variation (A) Molecular variation and epigenetic inheritance: frequency of variation generated during different molecular processes commonly seen in parasitic protozoans. Low frequency mutational events are heritable for much longer periods. Epigenetic variation maintains both a medium level frequency of variation and heritability and is ideally suited to respond to changes occurring within a transmission cycle. (B) Antigenic variation. Malaria parasites and African trypanosomes cause relapsing bloodstream parasitaemia in mammals. This is due to waves of parasites expressing different surface molecules (different colors): *P. falciparum* PfEMP1 molecules and *T. brucei* variant surface glycoproteins (VSGs). These VSGs are highly immunogenic, typically triggering an effective and lasting immune response, although immunosuppression can occur later during infection. The populations present at any time point can in fact be far more complex than shown and may contain large numbers of different clonal variants.

*P. falciparum* exports hundreds of proteins in the infected RBC (Hiller et al., 2004, Marti et al., 2004), dramatically transforming the cell and its surface with numerous proteins, including adhesins that facilitate sequestration to endothelial surfaces to avoid clearance of infected RBCs by the spleen. The main protein involved in cytoadhesion is *P. falciparum* erythrocyte membrane protein 1 (PfEMP1), which is encoded by the highly polymorphic *var* gene family of ∼60 virulence genes. Through the clonal and variant expression of single members of the *var* gene family, PfEMP1 variants are expressed one at a time, in a mutually exclusive fashion, facilitating antigenic variation (Scherf et al., 1998) (Figure 1). Ectopic recombination and immune selection has resulted in a remarkable diversity in these genes. The expression of a subset of PfEMP1 variants is associated with severe disease, as they bind specific host receptors, resulting in tropism for the brain or the placenta (Sampath et al., 2015, Turner et al., 2013).

Several other gene families have demonstrated antigenic variation in *P. falciparum* (Rovira-Graells et al., 2012, Witmer et al., 2012), including parasite ligand families required for RBC invasion (Coleman et al., 2012, Jiang et al., 2010) and proteins on the surface of infected RBCs that bind to uninfected RBCs in a process known as rosetting (Niang et al., 2014). Analysis of clonal lines of *P. falciparum* has indicated that variant expression occurs in many other multi-gene families involved in parasitic processes beyond antigenic variation, the transformation of the RBC during growth, transcriptional regulation, and lipid metabolism (Rovira-Graells et al., 2012).

African trypanosomes proliferate in the mammalian host bloodstream and, if a patient is untreated, will invade the CNS, which is ultimately lethal. These *T. brucei* parasites also have a complex life cycle with multiple proliferating and non-proliferating developmental stages. Morphological changes and major changes in energy metabolism and surface architecture occur when trypanosomes transition from the mammal to the insect host and also when parasites migrate from the tsetse fly mid-gut to the salivary gland. *T. brucei* cells also undergo considerable morphological changes while in the bloodstream, including the transition from proliferative slender forms to non-proliferative stumpy forms pre-adapted for transmission to the fly.

The extracellular environments encountered by *T. brucei* are quite distinct from the intracellular environments encountered by other pathogenic trypanosomatids like *Trypanasoma cruzi*, and by *Plasmodium* parasites. Indeed, *T. brucei* do not appear to enter host cells at any point during their life cycle. As an adaptation to cope with this extracellular lifestyle, where innate and adaptive immune attack is likely the greatest challenge, bloodstream-form *T. brucei* are coated with a dense layer of a super-abundant variant surface glycoprotein (VSG). The *VSG*s are subject to epigenetic control (Horn, 2014) and, like *var* genes, are typically clustered at subtelomeres (Berriman et al., 2005), in polycistronic *VSG* expression sites (*VSG*-ESs). Approximately twenty *VSG*s are linked to promoters that, in this case, recruit RNA polymerase I and are therefore competent for expression, but only one is expressed at a time. *VSG*-ESs contain up to twelve ES-associated genes that encode variant and surface-exposed transferrin receptors (*ESAG6/7*), adenylate cyclases (*ESAG4*), and folate transporters (*ESAG10*), proteins that likely facilitate the establishment and maintenance of an infection. The trypanosome surface is an attractive potential “point-of-attack.” Indeed, the VSG coat also accommodates other surface receptors and channels, either known or suspected to be involved in nutrition and metabolic control (Schwede et al., 2015). Genomic and other approaches are also now revealing many more African trypanosome surface proteins operating at the host-parasite interface (Jackson et al., 2010). In insect stage cells, the VSG coat is replaced by a procyclin coat consisting of EP and GPEET proteins that contain characteristic amino acid repeats, with “early” procyclic forms exhibiting a form of “social motility” that may be important for colonization and migration in the fly (Imhof et al., 2014), although, remarkably, the procyclins themselves are not required for colonization of the mid-gut.

### Silencing Multigene Families

#### Silencing the *var* Genes of *P. falciparum* in Heterochromatin

**Figure 2:**
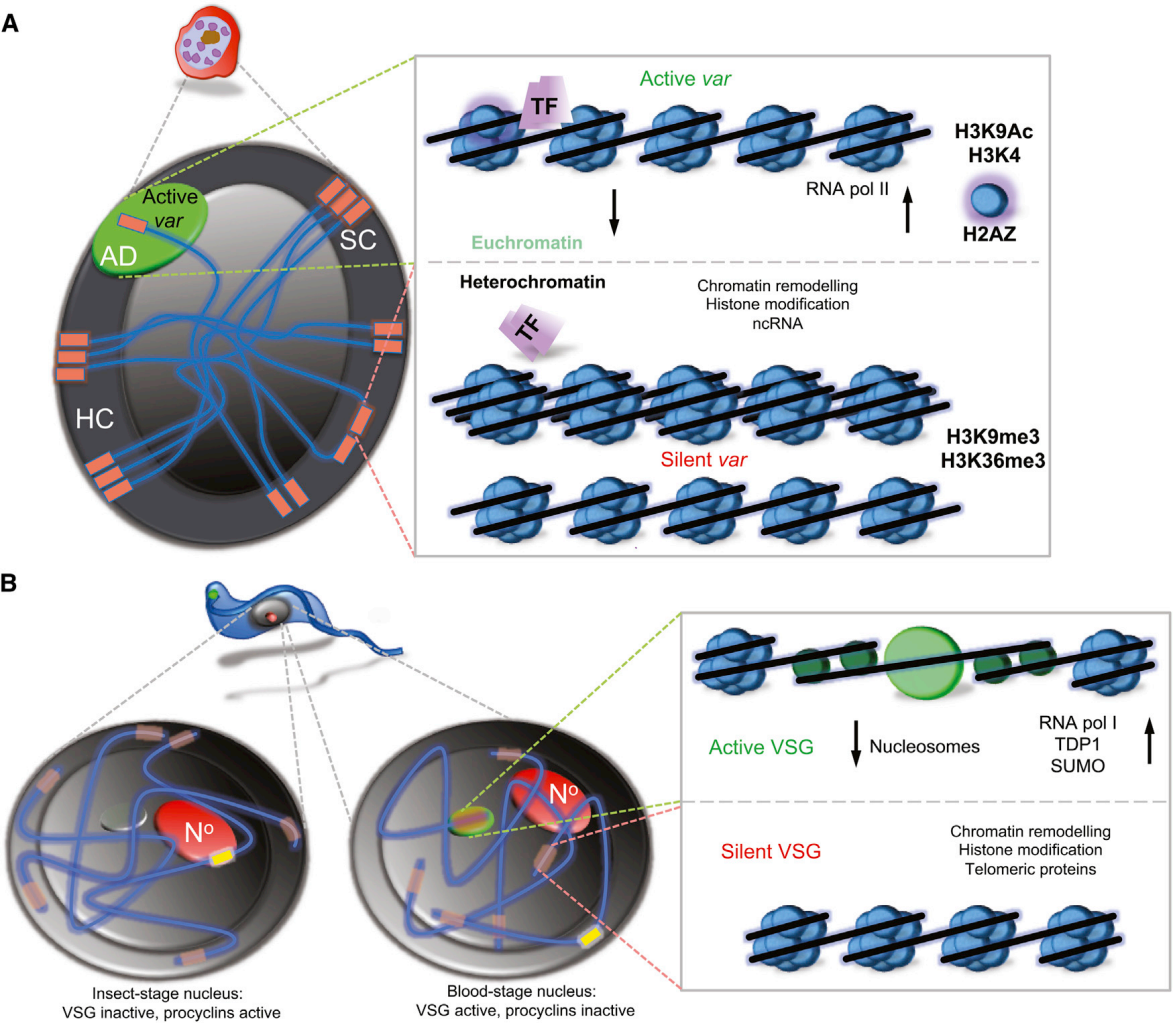
Nuclear Architecture and Locus-Specific Changes for Allelic Exclusion in *P. falciparum* and *T. brucei* (A) The nuclei of *P. falciparum* parasites (left panel) contain 14 chromosomes (only seven are shown) with *var* genes (orange, only a subset of the ∼60 genes are shown) found at all telomeres, and in internal tandem arrays. All *var* genes (sub-telomeric and internal) are localized at the nuclear periphery. Silenced *var* genes are found together in clusters of four to seven chromosome ends. Each nucleus normally has a single active *var* gene that is in a distinct domain at the nuclear periphery. Right panel: Transitions between an active and silent state for a *var* gene are associated with change at the locus from euchromatin to heterochromatin, associated with differences in the enrichment of specific histone modifications, the histone variant H2A.Z, and ncRNA. The active *var* gene is accessible to specific transcription factors, and the *var* gene is transcribed by RNA polymerase II. (B) An African trypanosome nucleus (left panel) contains eleven pairs of diploid chromosomes (only three shown) with VSGs (orange) at most, maybe all, telomeres. A single telomeric *VSG* is active in bloodstream-form cells and sequesters an extranucleolar pool of RNA polymerase I (green); a larger pool of pol I is sequestered at the nucleolus (N^o^). *VSG*s and *procyclins* (yellow) locate to the nuclear periphery in the stages in which they are inactive. Right panel: In bloodstream-form cells, the active *VSG*-ES is depleted of nucleosomes (blue) but enriched with a high mobility group protein (TDP1, dark green), RNA polymerase I (light green) and SUMOylated proteins.

In sequential generations of a clonal line of *P. falciparum*, most parasites will express the same *var* gene/PfEMP1 variant; however, at a very low switch-rate (∼0.1%–2%) (Frank et al., 2007), the formerly active *var* gene will become silenced, concomitant with the activation of a new *var* gene, resulting in antigenic variation. Genome sequences have provided a roadmap toward understanding the chromosomal organization of *var* genes in *P. falciparum*. Almost all *var* genes fall into three subgroups based on sequence similarities of their promoter regions: upsA, upsB, and upsC. Subgroups of PfEMP1 appear to possess different adhesive properties and switch-rates, suggesting that the expression of different members within each subgroup might represent different survival advantages. Recently, it was found that a subset of PfEMP1 with specific domains termed DC8 and DC13, encoded by upsA *var* genes, bind to endothelial protein C receptor (EPCR) within the brain, which could be a major factor in the pathogenesis of cerebral malaria (Sampath et al., 2015, Turner et al., 2013). Common to all of these genes is residence in subtelomeric regions or in tandem arrays, and all appear to localize to clusters at the nuclear periphery. These are perceived to be zones of silencing where repressive heterochromatin is formed (Figure 2A). In addition, epigenomic profiling has revealed at high resolution the distribution of histone modifications at a genome-wide level (Ay et al., 2014, Gupta et al., 2013, Hoeijmakers et al., 2013). It is clear that several histone marks are concentrated in the multi-gene families that encode virulence genes. This includes the H3K9me3 mark that is an evolutionarily conserved marker of heterochromatin (Lopez-Rubio et al., 2009) and the H3K36me3 mark (Jiang et al., 2013).

Many recent functional studies have determined that parasites require several enzymatic modifiers of histones, the writers and erasers of the histone code, for the silencing of all *var* genes (Table 1). These include a histone methyltransferase, PfSET2 (also known as PfSETvs) with a specificity for H3K36me3 (Jiang et al., 2013, Ukaegbu et al., 2014), as well as the histone deacetylase PfHda2 (Coleman et al., 2014). In addition, a central role can be attributed to the histone code reader *P. falciparum* heterochromatin protein 1 (PfHP1), whose depletion also results in a global deregulation of *var* gene expression (Brancucci et al., 2014). Indeed, in all of these cases, other genes that are enriched in the histone marks for heterochromatin (H3K9me3 and H3K36me3) were also largely deregulated, suggesting that that PfSET2, PfHda2, and HP1 are required for the formation and/or maintenance of heterochromatin.

Interestingly, the two members of the sirtuin histone deacetylase gene family, PfSir2a and PfSir2b, appear to silence alternative subsets of the *var* gene family, upsA and upsB *var* genes, respectively, suggesting that different *var* gene subsets are regulated through different silencing complexes (Duraisingh et al., 2005, Tonkin et al., 2009). This demonstrates a “division of labor” for epigenetic regulators as observed in model systems. PfSir2a is enriched at *var* promoters, and this binding can be overcome by overexpression of a domain of the origin replication complex protein (Orc1), suggesting a functional interaction (Deshmukh et al., 2012). Interestingly, depletion of PfRNase II, a chromatin-associated exoribonuclease enriched at silenced *var* genes, specifically removes the silencing of upsA *var* genes (Zhang et al., 2014), a phenotype similar to that observed following PfSir2a depletion (Duraisingh et al., 2005). These overlapping phenotypes following the perturbation of different epigenetic regulators suggest intimate functional interactions, either due to their presence in molecular pathways or even in protein complexes. PfSir2a-mediated effects on *var* gene were observed in one strain 3D7, and not in the strain FCR-3 (Merrick et al., 2015), suggesting the importance of genetic background and potential variation in the contributions of different epigenetic regulators.

Silencing complexes, as found in other eukaryotes, remain to be defined in *P. falciparum*. Recently, a bromodomain protein, PfBDP1, has been identified that is required for the silencing of genes involved in the invasion of RBCs by merozoites (Josling et al., 2015). Interestingly, PfBDP1 was found to be complexed with several other proteins including another bromodomain protein, PfBDP2. A comprehensive nuclear proteomic analysis has been carried out that should facilitate the identification of silencing complexes and many other aspects of nuclear biology in *P. falciparum* (Oehring et al., 2012). Future studies will be required to determine whether the effect of disruption of an epigenetic regulator on virulence gene silencing is due to a direct loss of enzymatic activity or due to disruption of a complex.

How heterochromatin is targeted to specific regions of the genome and not to others also remains an outstanding question. This could result from the action of DNA-binding domains with sequence specificity, such as members of the ApiAP2 family of DNA binding proteins. Interestingly, PfSIP2, an ApiAP2 protein that binds to specific sequence elements (SPE2) in the promoters of *var* genes, actually promotes the formation of heterochromatin and *var* gene silencing (Flueck et al., 2010).

#### Silencing the *VSG* Genes of *T. brucei*

The mechanism of *VSG* allelic exclusion, like that in malaria parasites, is not fully understood, but there are some common themes, in particular in terms of the requirements for gene silencing. Although *VSG*-ESs localize to the nuclear envelope and appear to form constitutive heterochromatin in insect midgut-stage cells (Figure 2B), they localize to the extranucleolar nucleoplasm in bloodstream-form cells (Navarro et al., 2007), possibly reflecting competence for expression and enhanced competence for recombination in this life cycle stage. The antigenic variation mechanism operating in the bloodstream-form African trypanosome, as in malaria parasites, requires the silencing of all but one variant gene. A role for repressive heterochromatin is clearly a common theme here. In the case of the *T. brucei VSG*s, nucleosomes are specifically depleted from the single active polycistronic *VSG*-ES (Figueiredo and Cross, 2010, Stanne and Rudenko, 2010), while knockdown of histone levels relieves silencing at inactive sites; histones H1 (Pena et al., 2014, Povelones et al., 2012) and H3 (Alsford and Horn, 2012) have been tested.

In addition to core chromatin components, facultative chromatin components and histone modifications also play a role (Table 1). A subtelomere-enriched histone H3-variant, H3.V, has recently been shown to have an impact on *VSG* silencing (Reynolds et al., 2016, Schulz et al., 2016), and multiple factors that either post-translationally modify histones, the writers and erasers, or bind modified histones, the readers, also have an impact. The dispensable histone tri-methyltransferase DOT1B that targets H3K76, for example, is required for rapid *VSG*-ES silencing and for an efficient transition from the active to the silenced state (Figueiredo et al., 2008). More recently, DOT1B was also shown to be responsible for spreading the silent domain from an initiation site at the *VSG* (Batram et al., 2014). Notably, DOT1A, the H3K76 di-methyltransferase, is required for mitotic checkpoint control in bloodstream-form *T. brucei* (Janzen et al., 2006), and DOT1B is required for karyokinesis in the first cell division during differentiation to the insect stage (Dejung et al., 2016). The histone deacetylases, DAC1 and DAC3 (Wang et al., 2010), and the acetyl-lysine binding proteins, BDF2 and BDF3 (Schulz et al., 2015), also have an impact on subtelomeric gene silencing, but the sites or subtelomeric regions they modify and bind, respectively, are not yet known; BDF3 is known to bind transcription start sites at chromosome-internal loci. A feature that appears to contrast to the situation in malaria parasites is that the sirtuin histone deacetylase, SIR2, although involved in telomeric silencing in *T. brucei*, has little to no impact on *VSG* silencing, presumably because SIR2 silencing is restricted to a small domain immediately adjacent to the telomere in *T. brucei* (Alsford et al., 2007). A bloodstream-stage-specific modified DNA base, J or hydroxymethyluracil found at silent *VSG*-ESs, and the factors responsible for its synthesis have also recently been shown to have a modest impact on *VSG*-ES silencing (Reynolds et al., 2016).

Factors that organize chromatin and its structure are also required for *VSG*-ES silencing (Table 1), including the histone chaperones, FACT (Denninger et al., 2010), ASF1 and CAF1 (Alsford and Horn, 2012), the ISWI chromatin remodeler (Stanne et al., 2015), and the trypanosome nuclear lamin, NUP1 (DuBois et al., 2012). Defects in DNA replication and chromosome segregation also lead to loss of silencing at *VSG*-ESs, as demonstrated by perturbation of origin recognition complex components (Benmerzouga et al., 2013), a mini-chromosome maintenance (MCM)-binding protein involved in the initiation of DNA replication (Kim et al., 2013) and cohesin components that link sister chromatids together (Landeira et al., 2009). Since many silent *VSG*s are subtelomeric, telomeric silencing in other cell types suggests that telomeres are also likely to be important for organizing silent chromatin in *T. brucei*. In support of this view, several telomere-binding proteins contribute to *VSG* silencing, RAP1 in particular (Yang et al., 2009), but also TRF2 (Jehi et al., 2014a) and TIF2 (Jehi et al., 2014b).

A recent study links inositol phosphate signaling to *VSG* expression control (Cestari and Stuart, 2015). In this study, knockdown of phosphatidylinositol 5-kinase or telomere-associated phosphatidylinositol 5-phosphatase, or overexpression of glycosylphosphatidylinositol (GPI) phospholipase C, derepressed silent *VSG*-ESs, and in the case of the kinase, triggered *VSG* switching. The VSG is connected to the surface membrane by a GPI anchor, so this may reflect communication between the cell surface and epigenetic machinery operating at the telomeric *VSG*-ESs. It should be noted here that in many instances above derepression phenotypes are moderate, despite substantial loss of viability, suggesting that there is more to allelic exclusion than gene silencing.

### Activation and Switching of Virulence Genes

While the vast majority of virulence genes are silenced, a single gene is expressed to carry out a specific parasitic function, also known as singular choice. While many regulators that are required for silencing have now been identified, the molecular basis for the selective activation of a single virulence gene remains mysterious. Recent studies have enigmatically defined specific features of the cellular architecture associated with activated virulence genes.

#### An Activation Domain at the *P. falciparum* Nuclear Periphery for *var* Gene Expression

**Figure 3:**
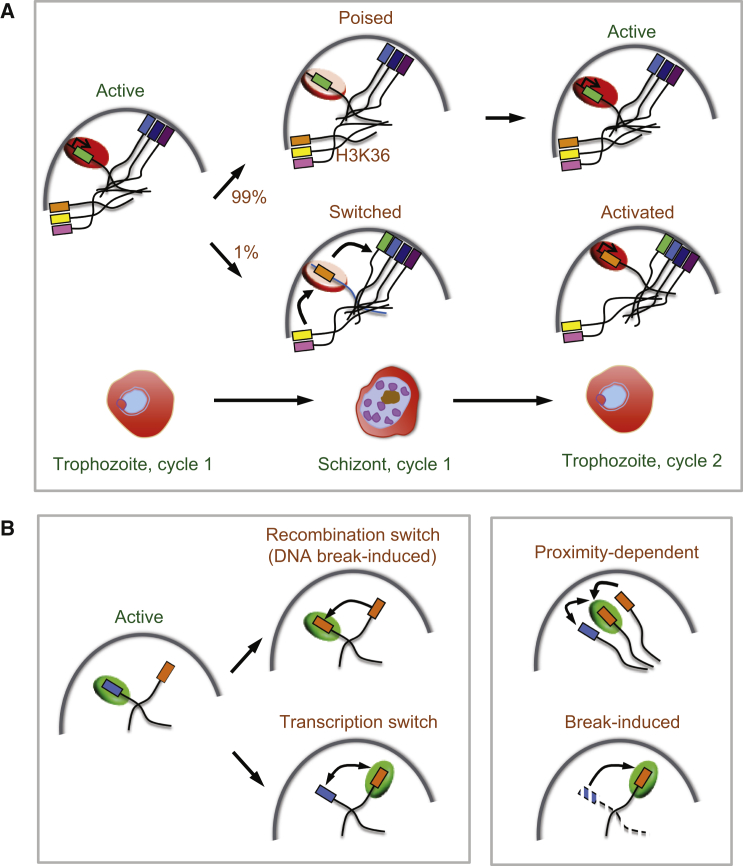
Locus-Specific Switching in Malaria Parasites and African Trypanosomes (A) Switching between silenced and active forms in *P. falciparum*. In ring-stage *P. falciparum* trophozoites, a single *var* gene is expressed from an activation domain at the nuclear periphery with histones enriched in H3K9Ac. At the time of nuclear division, the *var* gene that will be active in the following cycle remains in an activation domain in a “poised” state, with histones enriched in H3K4me3. At a low frequency (∼0.1%–2%), a new *var* gene is activated while the previously active *var* gene becomes silenced. *Var* genes that are deeply silenced between generations in heterochromatin contain histones enriched in H3K9me3 and H3K36me3. (B) Switching genetic and epigenetic states in *T. brucei*. Left panel: Extranucleolar RNA polymerase I (green) accumulates at the site of the active *VSG* (blue). A silent *VSG* (orange) can be duplicated and activated, replacing the active *VSG* (top panel) or by competing for pol I (lower panel). Right panel: Two features are shown that may impact these switching mechanisms; proximity may increase the probability of recombination or transcription switching (top panel), and a DNA break at the active site may liberate pol I, increasing interactions with other *VSG*s.

Analyses using FISH have identified all silenced *var* genes localized together in several clusters at the nuclear periphery (Freitas-Junior et al., 2005), while Hi-C profiling, which assesses the spatial arrangement and conformation of genomes, also suggests that all *var* genes are in close proximity to each other (Ay et al., 2014, Lemieux et al., 2013). The histone mark H3K9me3 associated with silent *var* genes co-localizes with the CENP-A marker associated with heterochromatin in model systems (Volz et al., 2010). Surprisingly, H3K9me3 appears to be concentrated in single foci, not in obvious clusters (Dahan-Pasternak et al., 2013). In *P. falciparum*, transcription of a single *var* gene occurs at a specific locus at the nuclear periphery, where the activated gene moves away from the silenced clusters (Figure 3A) (Duraisingh et al., 2005, Freitas-Junior et al., 2005). The cellular architecture supporting the active *var* transcriptional site is being elucidated. For example, the histone methyltransferase PfSET10 appears to co-localize with the transcriptionally active *var* gene. Interestingly, nuclear actin has been found complexed with PfSET10, which has been suggested to play a role in the movement of the locus during activation and heterochromatic silencing (Volz et al., 2012, Zhang et al., 2011). It appears that the activation domain usually only accommodates expression of one gene at a time. However, it can support the expression of at least two *var* genes (Brolin et al., 2009, Joergensen et al., 2010) as well as high numbers of *var* promoters (Dzikowski and Deitsch, 2008). Increased copy number of the *var* promoter results in silencing of all *var* genes, suggesting that activation of a single *var* gene is dependent on a limiting transcription factor (Brancucci et al., 2012). Similarly, promoters of other multigene families can also silence their family members, and transcription of members of the *rifin* family also co-localize to the activation domain (Howitt et al., 2009). It is unknown whether the activation domain is a discrete entity through which different chromosomes pass or whether the domain forms and is stabilized at the site of active transcription. Interestingly, the sub-telomeric rep20 repeat sequence appears to be sufficient for recruitment to the same domain as active *var* genes, suggesting the activation domain is a discrete nuclear structure (Duraisingh et al., 2005).

As with *var* gene silencing, *var* activation is associated with a specific histone code (Figure 2A), including the conversion from H3K9me3 to H3K9Ac, as well as trimethylation at H3K4 on histones, and the latter may be maintained by the action of PfSET10 (Volz et al., 2012). It has been postulated that PfSET10 maintains the active *var* gene in a poised state during schizogony, after it has been transcribed during the ring stage, which can epigenetically seed the inheritance of the *var* gene activation in the next asexual cycle (Volz et al., 2012). The poised state is characterized by the enrichment of H3K4me3, presumably through the activity of PfSET10.

*Var* gene promoters and introns require a one-to-one pairing for silencing, suggesting a complex interaction (Frank et al., 2006). The identification of “sterile” transcripts emanating from the intron has suggested a key role for long non-coding RNAs (lncRNAs) in the regulation of *var* genes. A bidirectional promoter within the intron produces transcripts that run into both neighboring exons, associated with chromatin at the nuclear periphery. Strikingly, the lncRNAs are associated with the single active *var* gene only when its promoter is active (Amit-Avraham et al., 2015), and a silent *var* gene can be activated by expression of its cognate lncRNA in *trans*. Removal of these lncRNAs resulted in a loss of *var* gene expression, epigenetic memory, and switching (Amit-Avraham et al., 2015). Analogy with other systems suggests that the lncRNA may direct the formation of chromatin with the loading of nucleosomes to propagate the activation state of a locus between generations. Indeed, active *var* gene promoters are also enriched with nucleosomes containing the histone variant H2A.Z (Petter et al., 2011). It remains to be seen whether the lncRNAs can recruit other key histone-modifiers to *var* gene loci.

#### Switching in the Expression of *va*r Genes

A picture that emerges for the ordering of events is as follows. In parasites that have survived the previous round of selection, the histone code is written onto new histones soon after synthesis. The *var* gene locus presumably transitions between heterochromatic and euchromatic regions of the nucleus in a stochastic fashion but with low frequency. Evidence suggests that this relocation is actin mediated (Volz et al., 2012, Zhang et al., 2011), and the frequency of the movement could vary in response to external stimuli. A silenced allele of the variantly transcribed invasion gene, PfRh4, can be forced out of the heterochromatic region shared with silenced *var* genes by activation of the neighboring gene; however, this does not automatically result in the complete activation of PfRh4 (Coleman et al., 2012). Instead, the frequency of activation of PfRh4 increases, suggesting that location alone is not sufficient for activation. With the transition of the locus from a heterochromatic to euchromatic region, activation presumably also requires the demethylation of H3K9, and the acquisition of the appropriate histone marks, followed by its stabilization as a euchromatic “poised” gene, (Figure 3A). In this scenario, the time spent in the heterochromatic or euchromatic zone being exposed to histone code writers/erasers will determine the on and off rate. The sum activity of these epigenetic regulators will result in post-translational modifications that describe a local histone code allowing readers such as PfHP1 and additional effectors to be recruited to the histone tails and seed the formation of heterochromatin. Once activated, *var* gene expression is maintained in successive generations in a clonal line.

In addition to the movement away from the silenced clusters at the nuclear periphery, it is clear that increased accessibility of the *var* gene promoter, as measured by hypersensitivity to micrococcal nuclease (MNase), is positively associated with activation (Duraisingh et al., 2005). This is analogous to the increased accessibility seen at the activated promoter of PfRH4, which also switches expression at high frequency (Coleman et al., 2012, Jiang et al., 2010). With increased accessibility, it is likely that sequence-specific DNA-binding proteins, such as members of the ApiAP2 family, bind to specific sequences in the *var* promoter and facilitate transcription.

In *P. falciparum*, both antigenic variation and sexual conversion occur at approximately 0.1%–2% per generation (Brancucci et al., 2015, Frank et al., 2007). Modeling suggests that this rate of switching is critical to allow antigenic variation to be successful in the long term in the face of the immune system (Coleman et al., 2014). However, the frequency of switching between *var* genes can vary dramatically between parasite strains (Fastman et al., 2012, Merrick et al., 2015). It also appears that the rate of switching differs dramatically depending on the *var* gene that is currently active (Enderes et al., 2011). The order may be related to different functions, such as cytoadhesion to specific niches within the body resulting from the expression of specific *var* gene subgroups. Members of upsA appear to be predominant in the host ex vivo, while in in vitro culture it is members of the upsB/upsC subgroups. An additional phenotype that has been little explored is the overall level of *var* transcription that also appears to be different between strains (Merrick et al., 2015). This suggests that there are significant differences in the ability of different *var* genes to remain activated, presumably due to differences in the machinery involved. Further, switching and the level of expression may be modulated by external factors both in vitro (Rosenberg et al., 2009) and in vivo (Merrick et al., 2012).

Evidence has now been obtained that the critical choice between asexual proliferation and sexual development leading to transmission in malaria blood-stage infections is epigenetically regulated. In *P. falciparum*, parasites persist within their human hosts in a state of asexual proliferation, with a small number of parasites converting into sexual forms. Recent evidence clearly demonstrates shared machinery between the regulators of antigenic variation and sexual development. Depletion of PfHP1 results in a dramatic conversion of asexual parasites toward sexual development (Brancucci et al., 2014) due to the activation of the *pfap2-g* gene that encodes a DNA-binding protein required for sexual conversion in both *P. falciparum* and the rodent malaria parasite *Plasmodium berghei* (Kafsack et al., 2014, Sinha et al., 2014). PfHP1 is found enriched at *pfap2-g* as well as at *var* genes. Clues to how specificity of a locus for silencing is determined may be provided by the observation that as *var* genes and the *pfap2-g* gene all have flanking insulator-like pairing element sequences (Avraham et al., 2012). Similar deregulation of both *pfap2-g* and *var* genes has also been observed following depletion of the PfHda2 histone deacetylase, identifying a potential upstream regulator of an epigenetic cascade with PfHda2 regulating PfHP1 for the switching between silenced and active states at specific genes (Coleman et al., 2014). Interestingly, depletion of PfSET2 does not result in activation of *pfap2-g* but does result in the deregulation of *var* genes.

It will be of great interest to identify the molecules that the parasite uses to sense changes in the environment and how these signals result in a switch to the sexual development program. PfHda2 and PfHP1 may be involved at different stages of the signaling cascade (Brancucci et al., 2014, Coleman et al., 2014). Interestingly, PfHda2 is expressed in the schizont-stage of the parasite when it could sense/transduce parasite signals. The timing of expression of PfHda2 allows it to act as a sensor of the environment by modulating its activity in the cycle before the rate of sexual conversion to gametocytes is altered. Epigenetic regulation is suitable for this switch as the change will be heritable for several cycles and can also be reversible in case the environment changes again.

Epigenetic switching on and off of transcription is the main mode of antigenic variation in *P. falciparum*, while recombination into expression sites as seen in *T. brucei* is rare. However, another layer of translational regulation exists in addition to epigenetic regulation for a specific *var* gene involved in placental malaria, var2csa (Bancells and Deitsch, 2013, Mok et al., 2008, Ukaegbu et al., 2015). Translation of a small upstream open reading frame (uORF) precludes translation of the downstream *var2csa* gene. It has been suggested that *var2csa* could play a central role in coordinating antigenic variation as *var* gene expression frequently defaults to this locus.

#### A Single *VSG* Expression Site Body in Trypanosomes

Recombination-based and transcriptional mechanisms can lead to *VSG* switching (Figure 3B, left-hand panel), but recombination makes a greater contribution to immune evasion in *T. brucei* compared to *P. falciparum*, and much research has therefore focused on DNA-repair in trypanosomes. The majority of *VSG*-switching events require RAD51-dependent homologous recombination, although microhomology-mediated end-joining also appears to make a substantial contribution (Glover et al., 2013). Indeed, the subtelomeric location of *VSG*-ESs is thought to favor recombination, since these domains are relatively unstable and prone to replication-fork collapse (Glover et al., 2013). *VSG*-ESs, present in multiple copies per genome, also harbor repetitive sequences, additional features that likely facilitate high rates of recombination.

In a clear parallel to the situation in *P. falciparum*, a large body of evidence supporting a role for telomeric chromatin in *VSG* gene silencing is now well established. Allelic exclusion, however, is more complicated and not so well understood. Exclusion requires coordination among gene-family members, with establishment and maintenance of distinct gene expression states. In *T. brucei*, a particular challenge is to understand how processive RNA polymerase I transcription is concentrated at one telomeric *VSG-*ES (Kassem et al., 2014) and how this can switch from one telomere to another (Figure 3B, right-hand panel). Indeed, a single extranucleolar focus of pol-I is present in bloodstream-form *T. brucei* and associates with the active *VSG*-ES (Navarro and Gull, 2001). These ESs segregate late during mitosis in a cohesin-dependent manner (Landeira et al., 2009) and, during the developmental transition to the insect stage of the life cycle, relocate to the nuclear periphery, concomitant with transcription inactivation and disassembly of the pol-I focus (Landeira and Navarro, 2007), consistent with conversion to a “closed” chromatin state (Figure 2B).

Progress has been made in this area recently, but a fundamental question remains. *VSG* exclusion could involve an active “default” state requiring negative control at all but one locus, or a silent “default” state requiring positive control at a single locus. Another alternative is a “default” state that is subject to coordinated activation or silencing. In particular, only modest derepression, typically observed following perturbations of numerous chromatin-associated factors, supports the latter scenario, which also invokes dedicated machinery for singular gene activation.

Although the active *VSG* is always adjacent to a telomere, suggesting that this location is important, no known telomere-binding protein has been found to be involved in *VSG* activation. The importance of chromatin structure and modification is also unclear, since nucleosomes are depleted at the active *VSG*-ES (Rudenko, 2010). A few factors associated with the active *VSG*-ES are implicated in activation, however. The high mobility group box protein, TDP1, appears to substitute for histones and to facilitate pol-I transcription at rDNA loci and at the active *VSG*-ES (Narayanan and Rudenko, 2013). Indeed, recent evidence suggests that TDP1 excludes nucleosomes and maintains the “open” chromatin structure at the active *VSG*-ES (Aresta-Branco et al., 2016). A feature that appears to be specific to the active *VSG*-ES is a focus of protein SUMOylation; although the SUMOylated factor(s) are not known, the SUMO ligase SIZ1 does appears to facilitate pol-I transcription (López-Farfán et al., 2014).

Although telomeres appear to be the only loci that naturally support *VSG* transcription, a *VSG* can be transcribed at a telomere-distal site (Batram et al., 2014). This was achieved using an engineered phage promoter and polymerase and was found to transiently interfere with native *VSG* expression. In another experiment revealing communication among *VSG*s, artificial blockade of the active *VSG*-ES allowed the high-frequency activation of other *VSG*-ESs (Aresta-Branco et al., 2016). These results are consistent with a remarkably robust exclusion mechanism operating in *T. brucei* and failure to select two stable, simultaneously active *VSG*-ESs (Chaves et al., 1999).

It can be a major challenge to determine whether a chromatin-associated factor is a cause or the consequence of a particular gene-expression status. For instance, are histones removed from *VSG*-ESs leading to activation, or does activation lead to histone removal? In this regard, it is worth considering allelic exclusion in the context of “directors” and “actors.” Based on this distinction, many conserved chromatin components and modifiers are likely actors, while specificity must be directed by other means (see Ptashne, 2013). As in *P. falciparum*, non-coding RNAs (ncRNAs) are excellent candidates that may confer specificity, but proteins are also likely involved. Remarkable examples of nucleotide-sequence programmable activities include the RNAi and the CRISPR-Cas9 machinery, and we wonder whether sequence programmable activities might also underpin allelic exclusion mechanisms in parasites and beyond.

#### VSG Switching Dynamics

Rates of antigenic variation are low in “experimental” *T. brucei* cultures, in the order of one switch per 10,000 to 1 million cells per generation. In contrast, recently fly-transmitted *T. brucei* have a much higher switch-rate, in the order of one switch per 500 cells per generation (Turner, 1997). High rates may reflect switching among or away from the subset of monocistronic subtelomeric *VSG*s active in the fly salivary gland; the distinct polycistronic *VSG*-ESs are activated in established bloodstream-form cells (Hertz-Fowler et al., 2008). The higher rates may also persist later in a bloodstream infection, and it has been suggested that high-frequency *VSG* rearrangement (Robinson et al., 1999), due to short telomeres (Hovel-Miner et al., 2012), is responsible. Regardless of the “natural” rate of switching, 30-day experimental infections in mice reveal complex mixtures of (∼30) variants at any time point (Mugnier et al., 2015), suggesting the need for de novo assembly of chimeric and novel *VSG*s to sustain a long-term infection (Hall et al., 2013, Mugnier et al., 2015).

Chromatin-associated “silencing-factors” have also been implicated in controlling the rate of *VSG* switching and antigenic variation, either through changing the frequency of switching transcription from one telomeric *VSG*-ES to another or through changing the frequency of successful recombination and repair, allowing new *VSG*s to replace the previously active *VSG*. The frequency of switching increases when cohesin components (Landeira et al., 2009) or the telomere-binding proteins, TRF2 (Jehi et al., 2014a) or TIF2 (Jehi et al., 2014b), are depleted, for example. In terms of *VSG* recombination, topoisomerase 3α (Kim and Cross, 2010) and the associated RMI1 subunit (Kim and Cross, 2011) are suppressive, and this may be necessary to maintain the integrity of the *VSG*-ES.

### Future Perspectives

**Figure 4:**
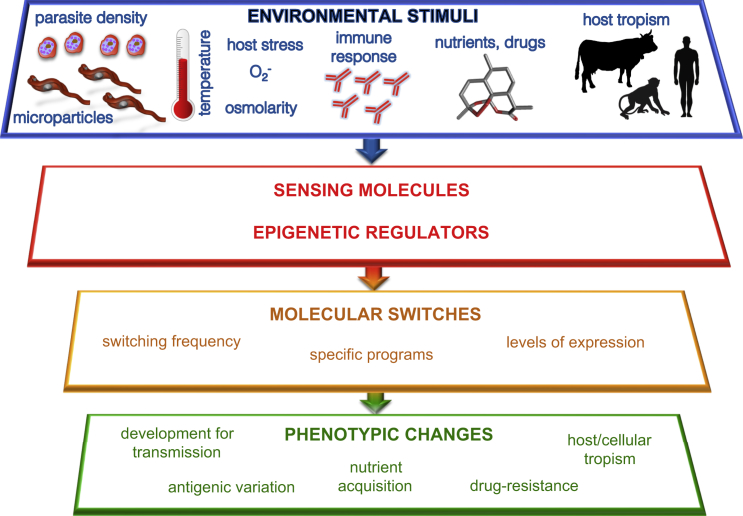
In Vivo Sensing and Epigenetic Regulation of Life Cycle Choices Parasites experience a variety of ecological niches during progression through their life cycles. Environmental signals are sensed by sensor molecules (including epigenetic regulators). These signals are integrated into decisions that are executed by the epigenetic machinery. These decisions can result in (1) different developmental programs being chosen, (2) differences in switch-rates for given choices, and (3) increases/decrease in the levels of specific virulence molecules and pathways. These transcriptional programs result in parasite processes, including choices between antigenic variation programs, sexual development, nutrient acquisition, and cellular tropism.

Epigenetic phenomena are increasingly recognized to be at the core of parasitic processes through their life cycles. Parasites demonstrate an amazing variety of life cycles, moving through diverse hosts, tissues, and cell types in their mammalian hosts and insect vectors. Indeed, the in vivo environment can profoundly affect the rate of switching. Environmental perturbations can include the development of an immune response, changes in the nutrition of the host, as well as host polymorphisms between hosts within a population. These all appear to be common challenges facing all parasites (Figure 4).

For the study of virulence gene expression, we need to further develop in vivo models and in vitro culture systems. Parasites may behave significantly differently in vivo, for example, due to particular host signals or features that are not effectively recapitulated in culture systems. Interactions with host immune effectors, and differences between host organs and compartments, are obvious examples.

Many fundamental mechanistic questions remain to be elucidated. Can we define different silencing complexes that regulate specific transcriptional programs? What is the nature and architecture of activation domains? What are the molecular determinants of singular choice? What are the epigenetic pathways for integrating diverse environmental signals? Increasingly powerful techniques will be employed to comprehensively measure post-translational modifications in various settings. For instance, DNA methylation has only recently been recognized in *P. falciparum* (Ponts et al., 2013).

It will also be important to understand which key regulators or interactions may be exploited for drug development. Proliferation and life cycle transitions could be targeted. For instance, the persistence of malaria parasite hypnozoite forms in the liver of vertebrate hosts could be eliminated through the use of drugs targeting epigenetic regulators (Dembélé et al., 2014). High-throughput screening assays will identify specific inhibitors of these processes that can also be used in chemical biology studies. A tangential translational approach would be the use of mutant parasites that are deleted in key epigenetic regulators, as vehicles for blood-stage vaccination, due to their expression of the complement of the virulence antigens due to failed allelic exclusion. Finally, a comparative biological analysis of related protozoan parasites could be particularly useful with regards to the recent evolutionarily pressures that have resulted in the species-specific elaborations observed.

Ten years ago, epigenetic regulation was a mysterious mechanism. An understanding of epigenetic phenomena is now revealing the remarkable capabilities of parasites to survive in a myriad of changing and often hostile environments as they are propagated through their life cycles. Significant progress has largely drawn on similarities with model systems. Also, the use of democratic genomic and functional genomic tools has led to the identification of the machinery involved. Powerful experimental systems are now in place for different parasites, and we are on the verge of a golden age, where mechanistic insights will be mapped to the processes of parasitism.

## Figures and Tables

**Table 1:** Epigenetic Regulators with Functional Evidence

Genes	Epigenetic Class	Mutation	Genes Affected	References
***Plasmodium falciparum***				

Hda2	Writer, class II HDAC	KD	Var,all; ApiAP2-G	Coleman et al., 2014
HP1	Reader, chromodomain	KD	Var,all; ApiAP2-G	Brancucci et al., 2014
SET2	Writer, KMT	KO	*Var*, all	Jiang et al., 2013
SET10	Writer, KMT	DN	Single *var* overexpression	Volz et al., 2012
PfRNase II	Exonuclease	KD	*Var*, upsA	Zhang et al., 2014
Sir2a	Writer, class III HDAC	KO	*Var*, upsA (3D7, not FCR3)	Duraisingh et al., 2005
Sir2b	Writer, class III HDAC	KO	*Var*, upsB (3D7, not FCR3)	Tonkin et al., 2009
BDP1	Reader, bromodomain	KD	Invasion	Josling et al., 2015

***Trypansoma brucei***				

BDF2	Reader, bromodomain	KO	VSG genes	Schulz et al., 2015
BDF3	Reader, bromodomain	RNAi	VSG genes	Schulz et al., 2015
DOT1B	Writer, KMT	KO	VSG genes	Figueiredo et al., 2008
FACT	Histone chaperone	RNAi	VSG genes	Denninger et al., 2010
ISWI	Remodeller	RNAi	VSG genes	Stanne et al., 2015
MCM-BP	DNA replication	RNAi	VSG genes	Kim et al., 2013
NLP	Remodeler	RNAi	VSG genes	Narayanan et al., 2011
NUP1	Nuclear lamin	RNAi	VSG genes	DuBois et al., 2012
ORC	DNA replication complex	RNAi	VSG genes	Benmerzouga et al., 2013
PIP5K	Inositol phosphate pathway	RNAi	VSG genes	Cestari and Stuart, 2015
PIP5Pase	Inositol phosphate pathway	RNAi	VSG genes	Cestari and Stuart, 2015
PLC	Inositol phosphate pathway	OverX	VSG genes	Cestari and Stuart, 2015
RAP1	Telomeric protein	RNAi	VSG genes	Yang et al., 2009
Siz1/PIAS1	Writer, SUMOylation	RNAi	VSG genes	López-Farfán et al., 2014
SUMO	SUMOylation	RNAi	VSG genes	López-Farfán et al., 2014
TDP1	Chromatin component	RNAi	VSG genes	Narayanan and Rudenko, 2013
H3.V	Chromatin component	KO	VSG genes	Schulz et al., 2016, Reynolds et al., 2016

HDAC, histone deacetylase; KMT, lysine methyltransferase; KO, knockout; KD, knockdown; OverX, overexpression; RNAi, knockdown; DN, dominant negative.
